# Surgical Site Infections in Mozambique: A Literature Review of Incidence, Antimicrobial Resistance, Risk Factors, and Surveillance Practices

**DOI:** 10.5334/aogh.5143

**Published:** 2026-03-02

**Authors:** Mahmood Yousry Mohamed El-Shazly, Rosa Buonamassa, Alessandro Cornelli, Ahmed Yousry El-Shazly, Roberta Iatta, Elmano dos Santos Gomonda, Luisa Frallonardo, Giacomo Guido, Mohamed El Shazly, Muhammad Asaduzzaman, Annalisa Saracino, Sónia Raquel Mendonça da Cunha, Raja Waqar Ali, Ferenc Balázs Farkas, Botond Lakatos, Francesco Di Gennaro, Ussene Hilário Isse

**Affiliations:** 1 Universidade Católica de Moçambique, Faculdade de Ciências de Saúde, Beira, Mozambique; 2 Department of Internal Medicine and Medical Therapy, University of Pavia (UniPV), Pavia, Italy; 3 Department of Mental Health and Public Medicine, Section of Infectious Diseases, University of Campania Luigi Vanvitelli, Via L. Armanni 5, 80131, Naples, Italy; 4 Department of Surgery, Hospital Central de Beira, Beira, Mozambique; 5 Interdisciplinary Department of Medicine, University of Bari, Bari, Italy; 6 Clinic of Infectious Diseases, Department of Precision and Regenerative Medicine and Ionian Area, University of Bari Aldo Moro, Italy; 7 Department of Gynecology and Obstetrics, Hospital Central de Maputo, Maputo, Mozambique; 8 Department of Community Medicine and Global Health, Institute of Health and Society, Faculty of Medicine, University of Oslo, Oslo, Norway; 9 Institute of Medical Microbiology, Faculty of Medicine, Semmelweis University, Budapest, Hungary; 10 Pediatric Center, Semmelweis University, Budapest, Hungary; 11 Semmelweis University Department of Internal Medicine and Hematology, Departmental Group of Infectious Diseases, Budapest, Hungary; 12 Minister of Health of Mozambique, Maputo, Mozambique

**Keywords:** surgical site infections, Mozambique, antimicrobial resistance, infection prevention, surveillance, low-resource settings

## Abstract

*Background:* Surgical site infections (SSIs) are among the most common healthcare-associated infections worldwide and impose a disproportionate burden in low- and middle-income countries (LMICs). In Mozambique, persistent health system constraints—including limited infection prevention and control (IPC) capacity, weak surveillance infrastructure, and rising antimicrobial resistance (AMR)—likely amplify this burden. This review synthesizes available evidence on SSI incidence, etiology, antimicrobial resistance patterns, risk factors, and surveillance practices in Mozambican healthcare settings.

*Methods:* A structured literature search was conducted in PubMed, Embase, Scopus, Web of Science, WHO Global Index Medicus, and Google Scholar for studies published between 2000 and September 2025. Eligible studies reported SSI incidence or prevalence, causative pathogens, AMR profiles, or associated risk factors in Mozambique. Additional data were retrieved from WHO reports, Joint External Evaluations (JEEs), and national surveillance assessments.

*Results:* Published evidence remains scarce and fragmented, with no comprehensive national estimates of SSI incidence identified. The most commonly reported pathogens were *Staphylococcus aureus* (including MRSA), *Klebsiella pneumoniae*, *Pseudomonas aeruginosa*, *Acinetobacter* spp., and *Escherichia coli*. MRSA prevalence in hospital settings ranged from 15% to 42%. Gram-negative isolates frequently demonstrated extended-spectrum β-lactamase (ESBL) production, suggesting substantial antimicrobial pressure. Reported risk factors were consistent with regional findings and included inadequate hand hygiene, suboptimal sterilization practices, prolonged lab or, malnutrition, HIV infection, and perioperative anemia. National SSI surveillance is largely absent, and only one hospital currently reports AMR data to the WHO Global Antimicrobial Resistance Surveillance System (GLASS).

*Conclusions:* SSIs represent a significant yet underrecognized public health challenge in Mozambique. Despite increasing multidrug resistance, systematic data collection and coordinated national surveillance remain limited. Strengthening IPC programs, establishing structured SSI surveillance, expanding microbiological laboratory capacity, and implementing antibiotic stewardship initiatives are urgent priorities to improve surgical outcomes and reinforce national health security.

## Introduction

Surgical site infections (SSIs) are infections that occur at or around a surgical incision within 30 days after an operation, or within 1 year when an implant is involved [1]. They represent one of the most frequent healthcare‑associated infections (HAIs) worldwide [1, 2]. SSI is also a leading cause of postoperative morbidity, prolonged hospital stays, increased healthcare costs, and patient deterioration in both high‑income and low‑ and middle‑income countries (LMICs) [3–5]. However, SSI is the most common HAI reported across hospitals in LMICs, with substantially higher risk than in high‑income settings [6, 7].

Across sub‑Saharan Africa (SSA), the rise of antimicrobial resistance (AMR) represents one of the most urgent public health threats and a critical barrier to safe surgical care. The African region bears a disproportionate share of global AMR‑related mortality, driven by limited microbiology capacity, weak surveillance systems, and widespread empirical antibiotic use [8–10]. Gram‑negative bacteria such as *Klebsiella pneumoniae*, *Pseudomonas aeruginosa*, and *Acinetobacter spp*. have shown resistance to multiple antibiotic classes, while methicillin‑resistant *Staphylococcus aureus* (MRSA) remains a dominant cause of postoperative infections in many African hospitals [11–13]. This growing resistance crisis worsens SSI outcomes by reducing therapeutic options, increasing complications, and prolonging hospitalizations—especially in settings with restricted access to second‑line antibiotics. In addition, several structural determinants contribute to the high SSI risk observed across SSA, including inadequate sterile processing, intermittent water supply, overcrowded wards and operating theaters, poor adherence to infection prevention and control (IPC) protocols, and the absence of systematic postdischarge surveillance—factors that collectively lead to underestimation of infection rates at the community level [8].

Mozambique, as a country in the SSA, provides a particularly relevant case study for understanding and addressing these systemic challenges. As a low‑income country with marked geographic and healthcare disparities, Mozambique delivers surgical care to a population with highly uneven access while many districts lack routine surgical services, and even where referral systems exist, multiple structural and socioeconomic barriers continue to limit access [14–16]. National risk assessments and Joint External Evaluations (JEEs) have repeatedly identified IPC programs as weak, inconsistently applied, and inadequately resourced. Although some tertiary hospitals have implemented basic SSI prevention measures, these remain neither standardized nor widely disseminated. Most importantly, there is a profound lack of research‑driven data and a reporting system for SSIs. Consequently, Mozambican surgeons and public health planners operate largely without reliable, nationally representative data on SSI incidence, etiologic agents, AMR patterns, or local risk factors.

It is therefore imperative to understand the system dynamics regarding SSIs and HAIs in countries like Mozambique for better public health management. This literature review aims to synthesize the available evidence as well as existing gaps on the epidemiology of SSIs and HAIs in Mozambique, with a specific focus on causative pathogens, AMR, risk factors, and prevention strategies, in order to bridge existing knowledge gaps and inform national policy and clinical practice.

## Materials and Methods

In this narrative review, we included articles published in English from January 2000 to September 2025. A comprehensive literature search was conducted using the following electronic databases: PubMed, Embase, Scopus, Web of Science, WHO Global Index Medicus (including the AFRO Library), and Google Scholar. The search strategy combined the keywords *Mozambique* AND (“surgical site infection” OR “nosocomial infection” OR “healthcare‑associated infection” OR “postoperative infection”).

To ensure completeness, we have manually screened the reference lists of all relevant articles to identify additional eligible publications. Supplementary information sources, including WHO reports, JEEs, and conference abstracts provided by the authors, were also examined to complement the peer‑reviewed literature.

Given the limited number of Mozambican studies available, the review followed a structured narrative approach, focusing on the extraction and synthesis of descriptive data related to SSI incidence or prevalence, etiologic agents, AMR, risk factors, and surveillance practices in Mozambican healthcare settings.

## Results

### Incidence

At the time of this review, no recent Mozambican study directly reported overall incidence rates of SSIs following surgical procedures. However, a systematic review [17] estimated a pooled prevalence of 11% for postcesarean section SSIs across Africa, with the highest rate observed in neighboring Tanzania (34.1%). Similarly, another review [18] reported an overall incidence of 10.5% for SSIs following orthopedic surgery in Africa, including a 6% incidence in Malawi. In Mozambique, Kynes et al. [19] identified that SSIs accounted for 2.7% of surgical deaths in 2015—substantially lower than the 23.1% reported by the GlobalSurg Collaborative [20].

### Etiology and antimicrobial resistance (AMR)

Clinical microbiology data from Mozambican hospitals highlight the growing threat of AMR. A Mozambican study [21] analyzing one year of positive cultures from the intensive care unit (ICU) of Maputo found that Gram‑negative bacilli accounted for approximately 79% of isolates, while Gram‑positive organisms represented the remaining proportion. The most frequently isolated pathogens included *Staphylococcus aureus*, *Klebsiella pneumoniae*, *Enterococcus spp*., *Acinetobacter spp*., *Pseudomonas aeruginosa*, and *Escherichia coli*. A substantial proportion of *S. aureus* isolates were methicillin resistant, while most Gram‑negative isolates exhibited extended‑spectrum β‑lactamase (ESBL) phenotypes, indicating significant antimicrobial pressure in hospital environments [22].

Consistent with broader SSA data, *S. aureus* (including MRSA) remains among the most common pathogens in wound, skin, soft‑tissue, and SSIs [21]. This was confirmed by a study conducted at a 728‑bed referral hospital in Beira between 2010 and 2011, which reported *S. aureus* in 51.1% of SSI and burn wound samples, with 15.1% being MRSA [23]. The same study found *S. aureus* to account for 89.3% of bacterial isolates in skin and soft‑tissue abscesses. Six years later, MRSA prevalence in the ICU of the same hospital had increased to 42.5% [24]. No evidence of broader AMR phenotypes among Mozambican SSI isolates has yet been reported.

### Risk factors

Risk factors for SSIs were not quantitatively analyzed in the Mozambican studies reviewed. However, available findings are consistent with those reported across SSA, identifying both process‑related and patient‑related contributors. Process‑related factors include poor hand hygiene compliance, inadequate sterilization procedures, and weak teamwork or safety culture in operating theaters [25]. Hospital‑level infection control failures have also been associated with the emergence of MRSA [26].

Broader determinants such as poverty, malnutrition, high prevalence of comorbidities (notably HIV and anemia), delayed presentation requiring emergency or contaminated surgery, multiple vaginal examinations before cesarean section, and inadequate staffing or sterilization infrastructure are likely to apply in the Mozambican context [6, 7, 27–29]. The absence of Mozambican multivariate studies examining associations between HIV status, nutritional deficiencies, and perioperative anemia remains a major gap in the national evidence base.

### Limited SSI surveillance data and insufficient IPC capacity

**Figure 1:**
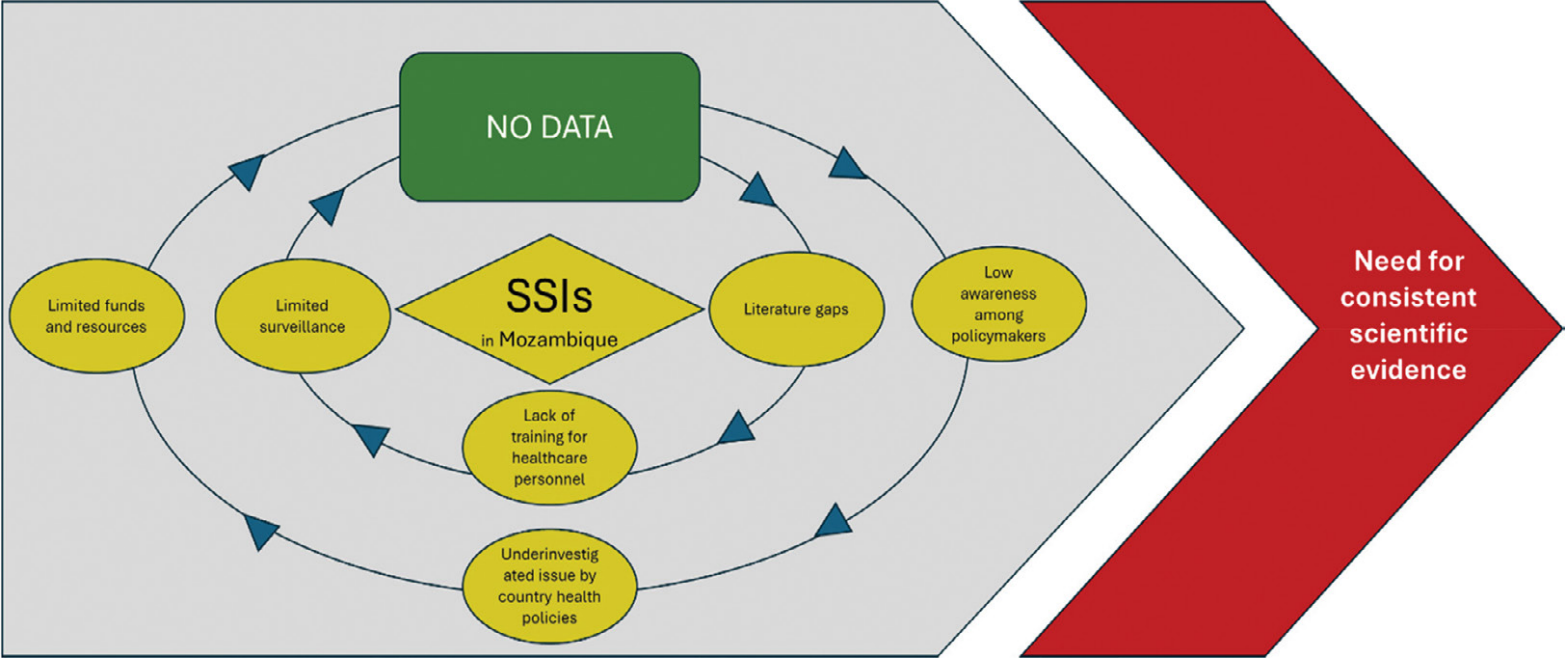
Cycle of evidence gaps and systemic barriers contributing to surgical site infections (SSIs) in Mozambique.

This review highlights both the considerable risk of SSIs in Mozambique and the profound lack of consistent, country‑specific data. As illustrated in Figure 1, the scarcity of reliable evidence creates a self‑perpetuating cycle in which limited surveillance, inadequate training of healthcare personnel, restricted funding, and low policymaker awareness contribute to the systematic underestimation of SSIs. These weaknesses hinder the development of appropriate prevention strategies and underscore the urgent need for robust scientific evidence to guide infection control interventions.

The figure illustrates how limited surveillance, insufficient IPC capacity, low awareness, and inadequate funding create a reinforcing cycle that perpetuates the underestimation and recurrence of SSIs across Mozambican hospitals.

## Discussion

The existing literature, summarized in Table 1, shows that Mozambique faces challenges similar to those encountered across SSA, where SSIs are aggravated by resource shortages, overcrowded wards, and inconsistent IPC practices [11]. Although some hospitals have implemented basic preventive measures—such as improved sterilization, antisepsis, and perioperative antibiotic prophylaxis—these initiatives remain fragmented, nonstandardized, and poorly monitored. The widespread emergence of resistant pathogens, particularly MRSA and ESBL‑producing Gram‑negative bacilli, further exacerbates this situation. The high resistance rates documented in neighboring SSA countries, combined with MRSA prevalence in Beira hospitals (15–42%) [21, 24], suggest that Mozambique faces a similar AMR burden [30, 31].

**Table 1:** Key recommendations for strengthening SSI prevention and control in Mozambique.

**Recommendations** A coordinated combination of system‑level improvements and targeted research should enable Mozambique to begin bridging the knowledge and control gaps in SSI. These recommendations require international cooperation and support from the WHO and NGOs to be implemented.		
1	Improve infection prevention by alleviating pre‑, intra‑, and postoperative practices.	This incorporates the implementation of WHO surgical safety checklists, strict hand and skin antisepsis, sterile technique, and operating room maintenance. Training and auditing should be increased, such as following the practices outlined in the compliance study and enhancing hand hygiene facilities.
2	Antibiotic stewardship	Revise national antibiotic prophylaxis guidelines to minimize broad‑spectrum use without compromising protection and to reduce unnecessary antibiotic administration after an operation.
3	Surveillance implementation	Implement a national SSI surveillance system. To begin with, SSI cases, as documented through a standardized definition, will need to be reported in sentinel hospitals, such as provincial referral centers, and submitted to Ministério da Saúde (MISAU). It is essential to train infection control teams to operate subcutaneous site monitoring, perhaps using CDC or WHO SSI event criteria. Mozambique will be able to experiment with surveillance at large hospitals and progressively apply it countrywide. Surveillance data will be used to describe the location of hotspots and assess the impact of interventions.
4	Laboratory capacity and AMR monitoring	Ongoing development of SSI pathogen network. Areas of laboratory capacity should be developed to enable the identification and susceptibility testing of SSI pathogens, as in GLASS work. This educates treatment and monitors the trends of resistance. Basic cultures of wound samples require an investment in lab reagents and staff training.
5	Address underlying risks	Due to high HIV and anemia in Mozambique, strong SSI prevention should include preoperative optimization, such as malnutrition and anemia, and prophylaxis in immunocompromised patients. Indirectly, SSI risk could be reduced by public health measures to control HIV, for example, by keeping people on ART.

Risk factors for SSIs have not been quantitatively explored in Mozambican studies; however, findings from the region point to both process‑related and patient‑related determinants. Poor hand hygiene, suboptimal sterilization, HIV infection, anemia, and malnutrition have been identified as major contributors [24, 25, 27]. The interplay between individual vulnerability and systemic fragility amplifies postoperative risk—particularly in emergency and obstetric surgeries [3, 32]. Furthermore, Mozambique’s national surveillance infrastructure remains at an early stage of development: only one hospital currently reports AMR data to the WHO Global Antimicrobial Resistance Surveillance System (GLASS), and SSI reporting relies almost exclusively on passive clinician notification [33, 34]. The absence of standardized definitions, trained IPC personnel, and active surveillance systems perpetuates under‑recognition of the problem and limits the allocation of financial and human resources [35–37].

Despite data limitations, several evidence‑based priorities emerge clearly from our current review. As outlined in Table 1, Mozambique must adopt a coordinated strategy combining system‑level improvements and targeted research to bridge knowledge and control gaps. Strengthening infection prevention throughout the pre‑, intra‑, and postoperative phases—through implementation of the WHO Surgical Safety Checklist, strict antisepsis protocols, and continuous staff training—remains essential. National antibiotic prophylaxis guidelines should be revised to rationalize antimicrobial use and minimize unnecessary postoperative antibiotic therapy. Establishing a national SSI surveillance system, beginning with sentinel hospitals, would allow standardized case detection and routine reporting to the Ministry of Health (MISAU).

Concurrently, laboratory capacity should be expanded to enable microbial culture and antimicrobial susceptibility testing, aligned with WHO GLASS recommendations. Preoperative optimization to address malnutrition and anemia, combined with tailored prophylaxis for immunocompromised patients, should be integrated into SSI prevention strategies.

## Operational Roadmap

To operationalize these recommendations, we have proposed an operational framework in Table 2 for strengthening SSI prevention and control in Mozambique, organized across five key domains: infection prevention, antibiotic stewardship, surveillance, laboratory capacity, and risk reduction [38]. Implementation of the WHO Surgical Safety Checklist and targeted IPC training at the facility level would establish the foundation for improved surgical safety culture. Revising national antibiotic prophylaxis guidelines and expanding antimicrobial stewardship programs would address the increasing burden of AMR and limit inappropriate postoperative antibiotic use. Establishing a national SSI surveillance network—starting with sentinel hospitals—and strengthening microbiology laboratories in alignment with the WHO GLASS initiative would generate essential data for clinical and policy decision‑making. Finally, integrating nutritional, hematological, and HIV screening into preoperative care would further mitigate infection risk.

**Table 2:** Operational framework for strengthening SSI prevention and control in Mozambique.

ACTION AREA	KEY INTERVENTIONS	LEAD ACTORS	REQUIRED RESOURCES	EXPECTED OUTPUTS
**Infection prevention**	Implement WHO Surgical Safety Checklists; strengthen hand and skin antisepsis; improve sterilization and operating room maintenance; regular IPC audits and staff training	Ministry of Health (MISAU); Hospital IPC Teams; WHO Country Office	Training materials, monitoring tools, supervision visits	Improved IPC compliance rates; reduction in intraoperative contamination
**Antibiotic stewardship**	Revise national surgical antibiotic prophylaxis guidelines; promote rational antibiotic use and reduce unnecessary postoperative prescriptions	MISAU; National AMR Committee; hospital pharmacy units	Technical expertise, local antibiogram data, stewardship training	Updated national prophylaxis policy; reduced broad‑spectrum antibiotic consumption
**Surveillance implementation**	Establish sentinel hospital SSI reporting network using standardized WHO/CDC definitions; develop electronic reporting to MISAU; regular feedback to facilities	MISAU; WHO; National IPC Program	IT infrastructure, data officers, IPC focal points	National SSI dataset established; periodic surveillance reports
**Laboratory capacity and AMR monitoring**	Strengthen microbiology laboratories to perform culture and susceptibility testing; integrate SSI pathogens into the national AMR surveillance system (GLASS)	National Laboratory Network; WHO GLASS Focal Point	Laboratory reagents, equipment, training	Functional AMR testing in sentinel hospitals; local antibiogram reports
**Address underlying risks**	Integrate screening and management of malnutrition, anemia, and HIV into preoperative care; reinforce ART and nutrition programs for surgical patients	Hospitals; NGOs; community health services	ART supply, nutrition supplements, outreach teams	Improved preoperative optimization; lower SSI risk among vulnerable patients

This operational framework will require multisectoral coordination, technical support from WHO and international partners, and sustained governmental commitment. By progressively implementing these actions over the next three years, Mozambique could develop a more resilient, data‑driven, and sustainable approach to surgical IPC.

Table 2 presents a phased operational roadmap for implementing SSI prevention and control priorities, combining system‑level improvements with patient‑centered strategies.

### Strength and limitations

The strengths of this review lie in its comprehensive synthesis of all available national and regional data, providing a structured overview of the current evidence gaps and challenges in Mozambique. However, certain limitations must be acknowledged. The number and quality of Mozambican studies are limited; no multicenter or longitudinal data are available; and potential publication bias may favor the reporting of successful interventions over routine surveillance findings. In addition, the reliance on English‑language publications and the extrapolation of findings from neighboring countries restrict the generalizability of conclusions.

## Conclusion

Surgical site infections remain an under‑recognized but significant public health challenge in Mozambique. We found substantial information scarcity over the past 25 years in Mozambique, evaluating incidence, surveillance strategies, risk factors, and the burden of AMR related to SSI. The combination of weak surveillance, limited laboratory capacity, and the emergence of resistant pathogens demands urgent action. Implementing WHO‑recommended IPC measures, expanding national surveillance and laboratory networks as per national action plan of AMR, and promoting antimicrobial stewardship are essential to strengthening Mozambique’s surgical safety system. Building a robust national evidence base through large‑scale, multicenter studies on SSI incidence, microbial resistance, and preventive interventions will ultimately inform more effective health policies, improve surgical outcomes, and enhance the country’s preparedness against infection threats. We believe that our current review will act as the guiding framework for further research and integrated actions in this domain.

## Data Availability

No primary data were generated for this study. All information used is derived from published literature and publicly available sources.

## Additional File

The additional file for this article can be found as follows:

Supplementary Table. Minimum annual resource needs per sentinel hospital for implementation of the IPC/SSI surveillance program.

This finding of multiple local introgression events has at least three key implications.
