# Supplementary material for: Surgical Site Infections in Mozambique: A Literature Review of Incidence, Antimicrobial Resistance, Risk Factors, and Surveillance Practices
Source: Ann Glob Health. 2026 Mar 2;92(1):24. doi: 10.5334/aogh.5143 (PMC12962243; doi:10.5334/aogh.5143)
Supplement: Supplementary Table. — Minimum annual resource needs per sentinel hospital for implementation of the IPC/SSI surveillance program. [file agh-92-1-5143-s1.pdf]

Supp Table. Minimum annual resource needs per sentinel hospital for implementation of the IPC/SSI surveillance program

Resource domain	**Component**	**Minimum requirement (per hospital/year)**	**Notes for cost translation**
Human resources	IPC nurse / focal point	0.5–1.0 FTE	Salary according to local public-sector scale
	Data officer / clerk	0.3–0.5 FTE	May be shared across departments
	Clinical staff time	Included in routine care	No additional FTE required
Training & capacity building	Initial IPC/SSI training	1 session	Trainers, materials, staff time
	Refresher training	1 session	Annual minimum
Surveillance system	SSI case reporting tools	Continuous	Paper or electronic
	Data reporting & feedback	Quarterly	Meetings or dashboards
Microbiology (mandatory)	Specimen collection	All eligible SSI cases	Swabs, consumables
	Culture capacity	Access guaranteed	On-site or referral laboratory
	Pathogen identification	Priority SSI pathogens	Basic identification
	AST	Routine for selected isolates	According to national protocol
	Quality assurance	SOP adherence	Internal QA minimum
IPC quality assurance	IPC audits	2–4 per year	Checklist-based
	Supervisory visits	0–1 per year	Regional/national level
Stewardship linkage	Use of local antibiogram	Annual update	Supports prophylaxis policy
Dissemination & coordination	Routine reporting	Quarterly	Facility → national level

IPC, infection prevention and control; SSI, surgical site infection; FTE, full-time equivalent; AST, antimicrobial susceptibility testing; AMR, antimicrobial resistance; WHO, World Health Organization; GLASS, Global Antimicrobial Resistance Surveillance System.
